# The Possibility of Replacing Wet-Milling with Dry-Milling in the Production of Waxy Rice Flour for the Application in Waxy Rice Ball

**DOI:** 10.3390/foods12020280

**Published:** 2023-01-06

**Authors:** Sicong Fang, Maoshen Chen, Feifei Xu, Fei Liu, Fang Zhong

**Affiliations:** 1 State Key Laboratory of Food Science and Technology, Jiangnan University, Wuxi 214122, China; 2 Science Center for Future Foods, Jiangnan University, Wuxi 214122, China; 3 School of Food Science and Technology, Jiangnan University, Wuxi 214122, China; 4 International Joint Laboratory for Food Safety, Jiangnan University, Wuxi 214122, China; 5 Jiaxing Institute of Future Food, Jiaxing 314015, China

**Keywords:** particle size, damaged starch content, physical properties, application quality

## Abstract

Due to the large consumption and discharge of water in wet milling, dry-milling is an alternative to produce waxy rice flour. The physical properties and sensory characteristics for preparing waxy rice balls in dry-milled waxy rice flour were compared in this study. The results showed that the damaged starch content increased significantly with the particle size of dry-milled flour, which decreased from 160 to 30 μm. The reduction in particle size increased the pasting viscosity of waxy rice flour, which further improved the stretch ability of dough and increased the viscoelasticity of the rice ball. The increase in damaged starch content directly led to a significant increase in the solubility of dry-milled flour, thereby increasing the freeze cracking rate of the rice ball and reducing its transparency, resulting in a decline in quality. In comparison with wet-milled waxy rice balls, dry-milled waxy rice balls made from rice flour in the range of 40 μm to 60 μm particle size had a similar texture and taste to that of wet-milled ones, moderate freeze cracking rate and better storage stability, as well as a stronger aroma of waxy rice that the consumer favored. GC-MS analysis showed that the content of key aroma compounds, such as grassy and fruity, noted nonanal in dry-milled flour, was 15–30% higher than that in the wet-milled depending on the difference of waxy rice variety. In conclusion, dry-milled waxy rice flour with a particle size in the range of 40 μm to 60 μm could be a candidate to replace wet-milled flour in the preparation of a waxy rice ball.

## 1. Introduction

A waxy rice ball is one of the traditional Chinese snacks, which is mainly made with waxy rice flour and water and usually stored at −4 °C. It has a round appearance with a diameter of about 2–3 cm, and has a soft and sticky taste [1]. It is generally believed that the waxy rice balls with complete appearance, high transparency (absorbance about 0.2 at 620 nm) and good viscoelasticity on taste has better quality. In addition, due to the influence of temperature fluctuations during freezing on the quality of waxy rice balls, waxy rice balls are required to have a certain storage stability, such as no frost cracks or collapses after freezing. These good qualities in waxy rice balls are highly related to the characteristics of the waxy rice flour, among which the particle size and damaged starch content of waxy rice flour are the most essential factors [2]. Studies have shown that the reduction of particle size can make the rice balls have a smoother surface and lower hardness (decreased from 526.0 g to 375.6 g) [3]. Another study showed that bread prepared with 106–150 μm had greater hardness and a rough taste while the bread prepared with a particle size of 75–100 μm had a suitable taste [4]. More importantly, the particle size and damaged starch content can directly determine the viscoelasticity taste of waxy rice balls after processing. According to Seigo et al. [5], the damaged starch content increased with the decrease of particle size, which further led to a significant increase in the solubility of waxy rice flour, making the higher pasting viscosity of waxy rice flour paste, which helped to improve the final taste of the product. However, the increase of solubility caused by the increase of damaged starch content [6] will reduce the transparency of soup after being cooked, thereby reducing the acceptability of consumers. As a consequence, waxy rice flour with a small particle size and low starch damaged content is often used to prepare high-quality waxy rice balls.

Due to the smaller particle size (10–40 μm) [7] and lower damaged starch content [8] in wet-milled flour, the wet milling method is widely used in the processing of waxy rice [9]. Despite the above advantages, the major concerns of wet milling are the huge sewage discharge that is generated during the soaking, crushing and screening process [10]. In addition, the water treatment in wet milling will cause the loss of some water-soluble substances of raw materials, and the drying process of materials will also correspondingly extend the production cycle and increase energy consumption [11]. Based on these problems caused by wet milling, it is urgent to find an alternative to the wet milling method. Therefore, it is proposed that we replace wet milling with dry milling for the above problems. However, previous studies found that the particle size of dry-milled flour is usually around 100–150 μm due to the limitation of machines, and it has a high content of damaged starch content [12,13]. This led to the poor viscoelasticity and rough taste of waxy rice balls prepared with dry-milled flour [14], which is not popular with consumers. Now, with the advancement of the industry, it is possible to prepare dry-milled flour with a smaller particle size [15], and the flour with a particle size similar to that of wet-milled can be obtained through further sieving. However, it is unclear which particle size of dry-milled flour is appropriate for preparing waxy rice balls, and how the increase of damaged starch content caused by the decreased of particle size will affect the quality of waxy rice balls still remains a factor to be further studied. Furthermore, understanding whether there are any other advantages in the application of dry-milled flour with a small particle size in waxy rice balls will help to develop the application potential of dry-milled flour.

The purpose of this study was to evaluate the characteristics of wet-milled flour and dry-milled flour with a small particle size in the application system of waxy rice balls, as well as to explore the impact of particle size on the quality of dry-milled waxy rice balls. The feasibility of preparing waxy rice balls with small particle size dry-milled flour instead of wet milled flour was analyzed, and the optimum particle size range of dry-milled flour for waxy rice balls was identified.

## 2. Materials and Methods

### 2.1. Preparation of Waxy Rice Flour, Dough and Waxy Rice Ball

Waxy rice from two cultivars, indica waxy rice (IWR) from Thailand and japonica waxy rice (JWR) from Taizhou in China, were prepared with wet milling and dry milling techniques, named IWR-W, IWR-D, JWR-W and JWR-D, respectively. In the dry milling method, waxy rice was milled with a high-speed universal crusher (SS-1022, Shengshun, Jinzhou, China) and then passed through a sieve with different meshes to the prepared IWR/JWR-D-number (sieve of mesh). The particle sizes are shown in Table 1.

Each dough was prepared by mixing flour with deionized water at 25 °C for 10 min. Different proportions of water were added to waxy rice flour according to the different characteristics of the sample. Wet-milled dough was mixed to 50% hydration (total water/dough, *w*/*w*) while the hydration of dry-milled dough was 42.9%.

A 6 g filling was wrapped with 10 g of dough and placed on a plate, then stored at −18 °C. A certain proportion of water (total water:rice balls, 10:1, *w*/*w*) was added to the pot containing the waxy rice balls. Then, they were cooked at 800 W for 5 min until the sample floated.

### 2.2. Particle Size and Damaged Starch Content of Waxy Rice Flour

The particle size of 0.5% waxy rice flour suspended in water was measured with a laser particle size analyzer (Malvern-3000, Malven Panalytical, Malvern, UK) and the average diameter of the volume (D(4,3)) of the sample was obtained. The damaged starch content was measured according to GBT9826-2008. In this method, the starch that is easy to hydrolyze with α-amylase is defined as damaged starch, and the measurement steps are as follows; 1.00 g (14% wet basis) flour sample and 0.050 g α-amylase were weighed in a 150 mL beaker. Then, the 45 mL acetic acid buffer solution (30 °C) was added and hydrolyzed at 30 °C for 15 min. After the reaction, 3.0 mL sulfuric acid solution (3.68 mol/L) and 2.0 mL sodium tungstate solution (0.4 mol/L) were added to terminate the hydrolysis reaction. The solution was filtered through filter paper, and the 5.0 mL of filtrate was put into the 50 mL tube, and the content of reducing sugar was determined according to GBT9826-2008.

### 2.3. Water Holding Capacity and Solubility of Waxy Rice Flour

The 0.5 g of waxy rice flour (dry basis mass, m) was put into a 50 mL centrifuge tube (m_0_), then 10 mL of deionized water was added and stirred for 1 h in a 95 °C water bath. Then, it was centrifuged at 12,000× *g* for 20 min; the supernatant (m_1_) obtained was dried in an oven at 105 °C, and the remaining sediment was weighed (m_2_). The solubility and water holding capacity were calculated with Equations (1) and (2), respectively.

(1) $$\mathrm{Solubility}(\%)=\frac{m_{1}}{m_{2}}\times 100$$

(2) $$\mathrm{Water}\;\mathrm{holding}\;\mathrm{capacity}(\%)=\frac{m_{2}-m_{0}}{m}\times 100$$

### 2.4. Pasting Properties

The pasting properties of waxy rice flour were tested using a rotational rheometer (Discovery DHR-3; TA Instruments, New Castle, DE, USA) according to Park et al. [16]. The sample (0.18 g) was weighed in a 5 mL centrifuge tube, and deionized water (1.3 mL) was added to prepare 12% (dry basis) rice flour dispersion. A parallel-plate geometry of 40 mm diameter was used and the gap was set to 1000 μm. The shear rate in all programs was 200 s^−1^ and the temperature program was set according to Fang et al. [17] as follows; each sample was held at 50 °C for 1 min, heated at a rate of 12 °C/min to 95 °C, maintained for 5 min and then cooled to 50 °C at a rate of 12 °C/min. A thin layer of silicone oil (Sinopharm, Shanghai, China) was added to the perimeter to prevent moisture loss during the measurement. The pasting viscosity was recorded.

### 2.5. Rheological Properties of Flour and Creep-Recovery Measurements of Dough

The viscoelastic properties of the sample was determined after the sample was completely gelatinized. After gelatinization in Section 2.4, the sample paste was kept at 50 °C for 300 s. Frequency sweep from 0.1 to 100.0 Hz was performed with a strain of 1%. The G′ and G″ were obtained and the tanφ value under 0.5 Hz were calculated.

Creep-Recovery test was performed on DHR-3 rhemeter equipped with a parallel plate (2000 μm gap, 40 mm diameter). The dough was kept for 120 s at 25 °C after loading. The creep test was measured at a shear stress of 50 Pa for 300 s, and then followed by a recovery phase of 360 s (shear stress 0 Pa). The creep-recovery parameters were obtained according to Filip et al. [18]. In the Creep-Recovery experiment, a certain shear stress (50 Pa) was applied to the dough sample, and the deformation degree of sample with the creep time was recorded. The maximum deformation of dough under the condition of applied stress was recorded as maximum creep strain (MCS). After the stress was removed, the maximum recovery deformation of the dough was recorded as the maximum recovery strain (MRS), and the recovery value (R) of the sample was calculated as follows:

(3) $$R(\%)=\frac{\mathrm{MCS}}{\mathrm{MRS}}\times 100$$

where MCS, MRS and R are the maximum creep strain, maximum recovery strain and % recovery.

### 2.6. Observation on the Gelatinization Process of Waxy Rice Flour

The gelatinization process of flour was observed with a polarizing microscope (DM 2700P, Leica AG, Wetzlar, Germany). The deionized water was added to prepare 0.1% sample suspension in the 5 mL tube. The sample was poured onto the center of a round glass slide and then placed on the hot stage of polarizing microscope. The overlapping glass slides was stick and seal using a special solid glue to prevent the overflow of waxy rice flour suspension. The sealed glass slide was put into the sample tank, and the temperature controller was connected. The temperature program was set as follows; the initial temperature increased from 25 °C to 95 °C at a rate of 2 °C/min and then cooled to 25 °C at a rate of 5 °C/s. In the process of temperature change, the images of waxy rice flour under the normal light were taken every 1 °C change to record the dynamic process of water absorption and swelling in the waxy rice flour granules. The magnification is 50 times, and the exposure time is controlled within 0.01 s.

### 2.7. Analysis of SPME-GC-MS

The adsorbed volatile substances of sample were extracted by headspace solid-phase micro extraction (HSSPME) and then transferred to a gas chromatograph/mass spectrometer (GC/MS) for analysis [19]. The 3 g sample was placed in a 30 mL headspace vial, and a volume of 4 μL of 1,3-dichloro-benzene (0.01306 mg/mL) was added to the vial as an internal standard. After 20 min of incubation at 60 °C, an extractor (CAR/DVB/PDMS) was inserted into the vial to adsorb the volatile organic compounds. 

All the volatile compounds were desorbed for 20 min at 230 °C and then separated on a DB-WAX column (30 m × 0.25 mm, df = 0.25 m, Agilent 123-7062, Santa Clara, CA, USA). The electronic energy on the mass spectrometer was 70 eV and the mass scanning range was 50~350 *m/z*. The temperature program settings were as follows: the sample was kept for 2 min at 60 °C, rose to 120 °C with 2 °C/min heating rate and kept for 2 min, then rose to 230 °C with 5 °C/min heating rate and kept for 4 min at 230 °C. The total duration of the procedure was 60 min. Finally, the NIST.17 spectral library was used for qualitative analysis of volatile compounds under the condition of matching degree >80. The limits of detection (LOD) and limits of quantitation (LOQ) of all compounds were within the determination range according to Zhu et al. [20].

### 2.8. Freeze Cracking Rate, Transparency and Appearance of Waxy Rice Ball

The freezing cracking rate of waxy rice ball was measured under the normal storage conditions and after accelerated test, respectively. The accelerated test was conducted in freeze–thaw cycles. In a freeze–thaw cycle, the frozen waxy rice balls were taken out and thawed in an oven at 25 °C for 1 h, and then, they were placed in a −18 °C refrigerator for 23 h. The freeze cracking rate of the sample was measured after four freeze–thaw cycles. Under normal storage conditions, the prepared waxy rice balls were directly put into the −18 °C refrigerator, and the freeze cracking rate of waxy rice balls was determined after the same storage time. According to the folds and cracks on the surface of waxy rice balls, it could be roughly divided into three types: unfrozen cracked, incompletely frozen cracked and completely frozen cracked, of which incomplete freezing cracks were counted as 0.5. The freeze cracking rate was calculated as follows:

(4) $$A\;(\%)=\frac{n_{1}}{n}\times 100$$

where A, n_1_ and n are the freeze cracking rate, quantity of frozen cracked waxy rice balls and total number of waxy rice ball.

The transparency of samples was determined with a UV spectrophotometer (UV-2802, Shanghai, China). Deionized water was added to the soup after cooking and the total volume was 500 mL and then cooled for 20 min. The deionized water was used as control group, and the OD value was measured at a wavelength of 620 nm.

In order to compare the difference in appearance of cooked waxy rice balls prepared with different milling methods, the appearance of samples was photographed and recorded.

### 2.9. Sensory Evaluation and Consumer Test

A 15-point scale was used to evaluate the flavor and texture of waxy rice balls. The sensory descriptors and reference of waxy rice balls were determined through group discussion. The main indicators included hardness, cohesiveness, chewiness and the aroma of the waxy rice. The evaluation process was carried out by a professional sensory evaluation team of 10 people. The evaluation process was carried out in a sensory evaluation room with an indoor temperature of 25 °C.

The consumer tests were conducted in the sensory laboratory at Jiangnan University. Sensory acceptance of waxy rice balls was evaluated by 50 panelists regarding the texture and taste. Overall likings were collected through 9-point hedonic scales, which ranged from “extremely disliked” (score 1) to “liked extremely” (score 9) [21]. During the test, purified water and crackers were available for cleansing the palates between samples.

### 2.10. Statistical Analysis

All the experiments were tested in triplicate. The data was analyzed with variance analysis (ANOVA) using IBM SPSS Statistics Version 22.0 software and presented as the average value ± standard deviation, and all data was plotted by using Origin 2018. The principal component analysis (PCA) was performed with XLSTAT 2019 software, and GC-MS data was analyzed with Masshunter software.

## 3. Results and Discussion

### 3.1. The Changes in Physical Characteristics of Dry-Milled Waxy Rice Flour with Decreased Particle Size

In this study, the quality of waxy rice balls prepared by two cultivars of waxy rice flour were compared to prove the universality of experimental results. Two methods (wet milling and dry milling) were selected for sample treatment. The dry-milled flour with different particle size was obtained by sieving through screen with different apertures, which were uniformly named as IWR/JWR-D-number (the number is the mesh number of screen), such as IWR-60, IWR-120, IWR-200 and IWR-320, etc. It could be seen from Table 1 that the particle size range of D-60 was within 150–170 μm, the particle size range of D-120 was 80–100 μm and its D-200 was within 50–70 μm while D-320 had the smallest particle size among all dry-milled flour, within 20–40 μm. However, the particle size of wet-milled flour of all samples were still smaller than that of dry-milled flour, which was within 10–20 μm. It had the same particle size range as the wet-milled flour prepared by Leewatchararongjaroen et al. [7].

As the main raw material of waxy rice ball, the physical properties of waxy rice flour directly affect the quality of waxy rice ball. The key physical properties mainly include damaged starch content, solubility, pasting characteristics, etc. Therefore, it is necessary to further explore the physical properties of waxy rice flour obtained with different milling methods to explain the differences in the quality of waxy rice ball in the later stage.

#### 3.1.1. Damaged Starch Content, Solubility and Water Holding Capacity of Waxy Rice Flour

The damaged starch content of dry-milled and wet-milled flour was compared in Table 1. It could be seen that the damaged starch content of dry-milled flour in two cultivars increased significantly with the decrease of their particle size. In IWR, the damaged starch increased from 9.40% to 26.22% when the particle size was reduced from 164 μm (IWR-D-60) to 27.10 μm (IWR-D-320). The same trend was observed in JWR. Moreover, when the particle size of dry-milled flour decreased to be similar to that of wet-milled, the damaged starch content of dry-milled flour was significantly higher than that of the wet-milled (6.03% and 5.70%), and the trend was the same between two cultivars. The marked difference in damaged starch content between dry-milled and wet-milled flour was directly related to its milling process. The pre-soaking and water-adding in wet milling significantly reduced the hardness of waxy rice granules so that wet-milled flour with less damaged starch content could be obtained [22]. Although the dry-milled flour with smaller particle size could be obtained by repeating milling, it led to a corresponding increase in damaged starch content [8].

The solubility and water holding capacity of waxy rice flour after gelatinization were shown in Table 1. The results showed that the solubility increased with the decrease of particle size, such as increased from 34.89% to 42.03% in IWR, and increased from 51.02% to 56.16% in JWR. In addition, when the particle size of dry-milled flour was similar to that of wet-milled, their solubility was significantly higher than that of wet-milled flour. The solubility of D-320 were 42.03% and 56.16%, respectively, which were significantly higher than that of wet-milled (9.28% and 16.51%). The changes in solubility were highly correlated with the damaged starch content. It is well known that water molecules preferentially enter the incompletely structured starch molecules at gelatinization temperature, thereby improving the solubility of waxy rice flour [23]. Previous results in damaged starch content showed that the reduction of particle size would significantly increase the damaged starch content, which resulted in a higher solubility of dry-milled flour with a small particle size. In addition, it could be seen from Table 1 that the water holding capacity gradually decreased with the particle size decreased. The water holding capacity of IWR-D decreased from 8.79% to 7.91% and that of JWR-D decreased from 6.90% to 6.47%. There was a marked difference between dry-milled and wet-milled flour with similar particle size. The water holding capacity of IWR-W was 18.13% while that of IWR-D was only 7.92%. The sample with high solubility had lower water holding capacity; this indicated that the water holding capacity was negatively correlated with solubility, which was consistent with Lee et al. [7]. It could be concluded that damaged starch content will further affect the water holding capacity and solubility of waxy rice flour after gelatinization. Whether this difference will affect the quality of waxy rice balls remains a factor to be further studied.

#### 3.1.2. Pasting Properties of Waxy Rice Flour

**Figure 1:**
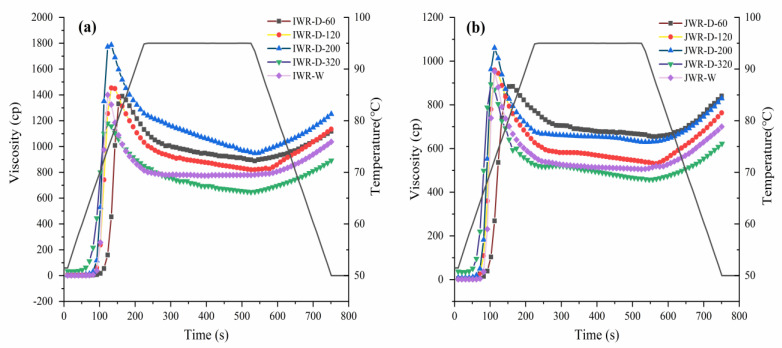
The pasting viscosity of waxy rice flour. (**a**) stands for the pasting viscosity of different particle size of IWR−D and IWR−W; (**b**) stands for the pasting viscosity of different particle size of JWR−D and JWR−W.

The pasting curves of waxy rice flour are shown in Figure 1. In the different particle size of dry-milled flour, the pasting viscosity increased first and then decreased with particle size reducing. The viscosity in two cultivars reached the maximum at D-200 but decreased sharply at D-320. Among them, IWR-D increased from 1453.75 cP to 1786.59 cP and then decreased to 1179.08 cP, and JWR-D increased from 959.17 cP to 1060.33 cP and then decreased to 894.30 cP. Moreover, when the particle size of dry-milled flour was similar to that of wet-milled, the dry-milled flour had lower pasting viscosity. The trend was the same between the two cultivars. Previous studies [6] showed that particle size and damaged starch content were important factors affecting the gelatinization behavior of waxy rice flour. When the particle size of dry-milled flour was between D-60 and D-200, particle size was the main factor affecting the pasting viscosity. The samples with smaller particle size were more likely to gelatinize, which led to an increase in pasting viscosity. However, when the particle size continued to decrease to D-320, the high content of damaged starch (26.22% and 24.64%, respectively) made the starch granules easier to break when swollen with water absorption, resulting in a sudden decline in pasting viscosity. Similar results were observed by Hossen et al. [24]; it was found that the damaged starch content increased significantly when the particle size was reduced to 10–20 μm, resulting in a significant decrease in its pasting viscosity. That is why the wet-milled flour still maintained a higher pasting viscosity in the same particle size range.

**Figure 2:**
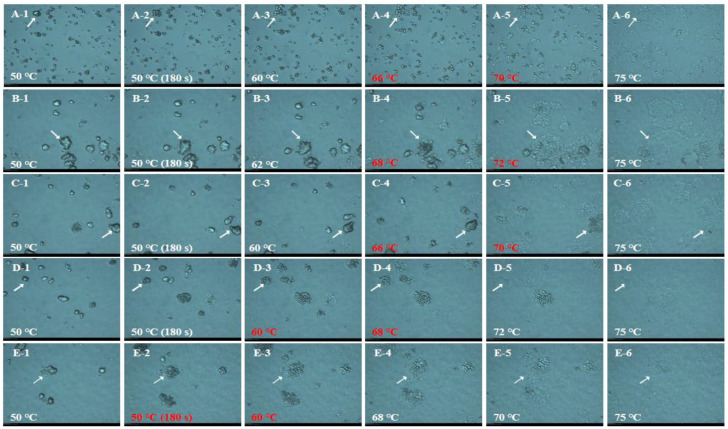
The dynamic gelatinzation process of IWR. “A−1–A−6”, “B−1–B−6”, “C−1–C−6”, “D−1–D−6” and “E−1–E−6” stand for the dynamic gelatinzation process of IWR−W, IWR−D−60, IWR−D−120, IWR−D−200 and IWR−D−320, respectively. The temperature represents the swelling temperature of the sample.

**Figure 3:**
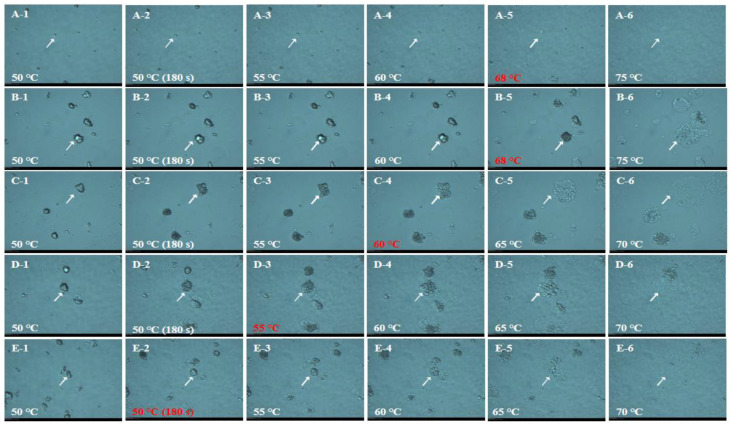
The dynamic gelatinzation process of JWR. “A−1–A−6”, “B−1–B−6”, “C−1–C−6”, “D−1–D−6” and “E−1–E−6” stand for the dynamic gelatinzation process of JWR−W, JWR−D−60, JWR−D−120, JWR−D−200 and JWR−D−320, respectively. The temperature represents the swelling temperature of the sample.

To further explore the effects of particle size and damaged starch content on the pasting properties of waxy rice flour, the dynamic gelatinization process was observed in Figure 2 and Figure 3. It could be seen that the gelatinization temperature decreased with a dry-milled flour particle size reducing. IWR-D dropped from 68–72 °C to about 50 °C, and the same trend was also observed in JWR-D. In the same particle size range, the swelling temperature of IWR-W and JWR-W were 66 °C and 68 °C, respectively, higher than that of dry-milled. This phenomenon indicated that the increase of damaged starch content would reduce the gelatinization temperature of system, thus accelerating the gelatinization process [25]. Therefore, particle size and damaged starch content jointly affect the pasting properties of waxy rice flour. The pasting viscosity of the system increased appropriately with particle size decreasing when the particle size was larger than 35 μm. While the damaged starch content was the key factor affecting the pasting viscosity when the particle size was less than 35 μm. With the increase of damaged starch content, the pasting viscosity decreased significantly.

#### 3.1.3. Rheological Properties of Waxy Rice Flour

**Figure 4:**
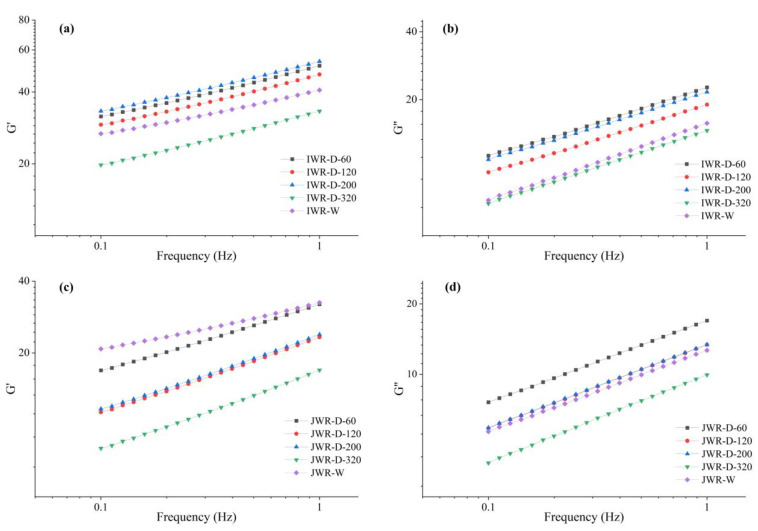
The viscoelastic properties of waxy rice flour. (**a**,**c**) stand for the storage moduli (G′) of IWR and JWR, respectively. (**b**,**d**) stand for the loss moduli (G″) of IWR and JWR, respectively.

Figure 4 demonstrated the viscoelastic properties of waxy rice flour paste. The storage moduli (G′) and loss moduli (G″) increased with the frequency in all samples, and the value of G′ was always larger than the value of G″, which indicated that the waxy rice flour paste tended to be elastic [26]. The value of phase shift tangent (tan) was obtained by calculating at 0.5 Hz, and the results were shown in Table 2. The tan of all samples were between 0.36 and 0.61, which indicated that the waxy rice paste behaved as weak gel [27].

It could be found that the tanφ value increased gradually with particle size decreased in IWR-D (from 0.372 to 0.415) and JWR-D (from 0.509 to 0.610), which represented that the elastic characteristics gradually weakened while the viscous properties increased. This was consistent with the previous results of pasting viscosity (Section 3.1.2). The reduction of particle size could improve the viscosity to a certain extent, but starch granules were more likely to rupture after swelling due to the corresponding increase in damaged starch content, which weakened the rigidity of system and reduced the elastic characteristics [28]. Therefore, the elastic characteristics of IWR-W and JWR-W with low damaged starch content were more obvious compared to dry-milled flour with the same particle size, and their tan values were 0.368 and 0.358, respectively.

### 3.2. The Changes in Physical Characteristics of Dry-Milled Waxy Rice Flour with Decreased Particle Size

#### 3.2.1. Waxy Rice Dough Stretch Ability

According to the difference of water holding capacity between dry-milled and wet-milled flour, the dough formula was determined as follows; the flour-to-water ratio for wet-milled dough was 1:1, and for dry-milled, it was 4:3. The dough for making waxy rice balls needs to have a certain stretching ability and shaping ability to wrap fillings more easily and maintain its shape during the freezing process [3]. The creep-recovery curves could characterize the deformation and stretching ability of the dough [29]. Therefore, a creep test at a shear stress of 50 Pa was performed, and the results were reported in Table 3.

Recovery value of samples could be used to measure the stretch ability of dough. With the particle size decreased, the recovery value of all samples increased significantly from 41.18% and 37.74% to 95.66% and 93.25%, respectively. This indicated that the reduction of particle size could significantly improve the deformation and tensile capacity of dry-milled dough. When the particle size was reduced to be similar to that of wet-milled, its recovery value reached 95.66% and 93.25%, respectively, which was significantly higher than that of wet-milled dough (38.89%). In addition, there was no significant difference in stretch ability of waxy rice dough between two cultivars. However, it was found that the IWR-W and JWR-W dough with high hardness and IWR-D-320 and JWR-D-320 dough with high viscosity greatly reduced the wrapping practicability in operation. On the contrary, IWR-D-200 and JWR-D-200 dough had moderate hardness, viscosity and stretch ability, which could wrap the fillings well.

#### 3.2.2. Freeze Cracking Rate of Waxy Rice Balls

Waxy rice balls are usually frozen at −4 °C, and the rice balls with good quality should have good storage stability. The quality of waxy rice balls is greatly reduced by the wrinkles or cracks caused by temperature fluctuation during storage, so the freeze cracking rate is a main indicator to evaluate the storage stability. It is necessary to predict the storage stability of waxy rice balls by measuring the freeze cracking rate after accelerated experiment [3].

**Figure 5:**
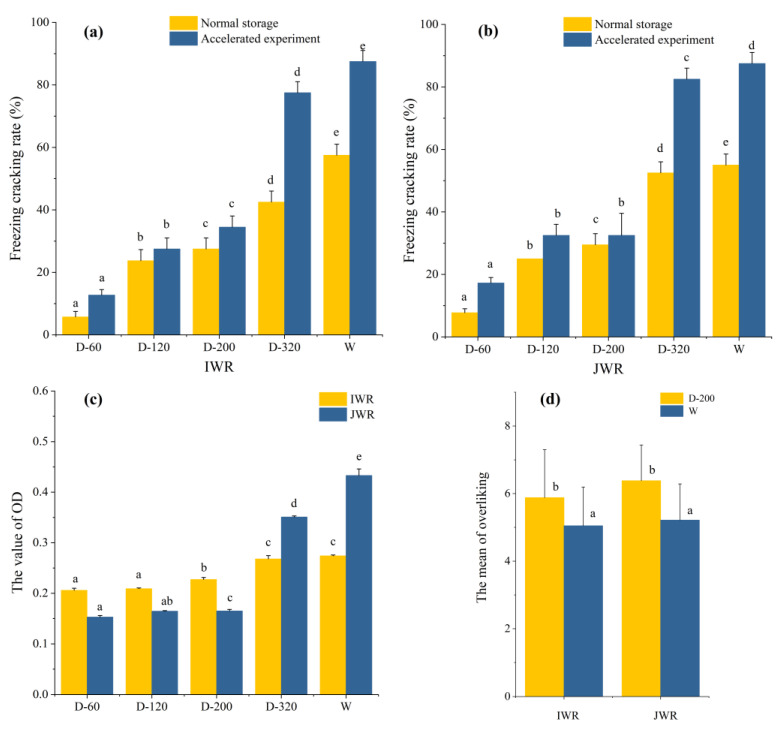
The indicators of waxy rice balls. (**a**,**b**) stand for the freezing cracking rate of waxy rice balls in IWR and JWR, respectively; (**c**) stands for the value of OD, the OD value was the absorbance of samples’ soup at 620 nm; (**d**) stands for the mean of overall liking in consumer tests. Different letters within a column indicate significant differences between mean values (*p* < 0.05).

It could be seen from Figure 5a,b that the freeze cracking rate increased with the decrease of particle size under the normal storage condition. The freeze cracking rate of IWR-D-60 and JWR-D-60 were the lowest (less than 10%) while that of IWR-D-320 and JWR-D-320 were as high as 50%. The results in Section 3.1.1 showed that the water holding capacity of dry-milled flour was reduced by particle size decreased, and waxy rice balls with lower water holding capacity were more likely to crack after freezing. Therefore, IWR/JWR-D-320 had the highest freeze cracking rate. However, the freeze cracking rate of IWR/JWR-D-320 was still lower than that of wet-milled rice balls with the same particle size. This may be related to the dough formula. More water was added to the wet-milled dough, resulting in a slower freezing speed under the same conditions, so the possibility of freezing cracking was greatly increased. After accelerated experiment, it could be observed that the freeze cracking rate of all samples had been improved. The freeze cracking rate of D-320 and wet-milled waxy rice balls increased significantly, reaching about 80% while that of D-60, D-120 and D-200 dry-milled waxy rice balls increased slightly. This indicated that D-60, D-120 and D-200 waxy rice balls had better storage stability.

#### 3.2.3. Appearance, Transparency of Soup and Texture Characteristics of Waxy Rice Balls

**Figure 6:**
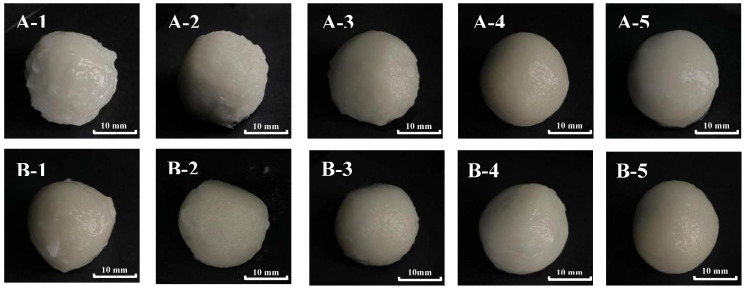
Appearance of waxy rice balls. A−1 stands for IWR−D−60, A−2 stands for IWR−D−120, A−3 stands for IWR−D−200, A−4 stands for IWR−D−320, A−5 stands for IWR−W; B−1 stands for JWR−D−60, B−2 stands for JWR−D−120, B−3 stands for JWR−D−200, B−4 stands for JWR-D−320, B−5 stands for JWR−W.

The appearance of waxy rice balls is one of the key factors that directly affects consumer evaluation. Therefore, the appearance of waxy rice balls prepared by all samples were recorded and shown in Figure 6. It was obvious that there was a marked difference between dry-milled and wet-milled waxy rice balls. The surface of wet-milled waxy rice balls were smooth, round and shiny while the surface of IWR/JWR-D-60 and IWR/JWR-D-120 samples had a “burr” phenomenon and looked rough. However, the surface of dry-milled balls gradually became smooth and glossy with the decrease in particle size. The appearance of IWR/JWR-D-200 and IWR/JWR-D-320 were more similar to that of wet-milled waxy rice balls. This indicated that reducing the particle size of dry-milled flour could significantly improve the appearance of dry-milled waxy rice balls. When the particle size was closed to that of wet-milled flour, the appearance of dry-milled balls was smooth and glossy.

In addition, the transparency of the soup is also an important indicator for evaluating, which could directly reflect the overall quality of waxy rice balls after cooking. The results were shown in Figure 5c. The OD value represented the turbidity of the soup. The higher the OD value, the lower the transparency of the soup. In dry-milled with different particle size, it was found that the OD value was increased from 0.209 to 0.268 (IWR-D-320) and 0.165 to 0.351 (JWR-D-320), respectively. This meant that the transparency decreased with the particle size reducing, which had a negative effect on the quality of waxy rice balls. However, the OD value of wet-milled flour (0.274 and 0.433) was still higher than that of dry-milled in the same particle size range. This phenomenon was related to the solubility of waxy rice flour. Previous studies showed that the flour with high solubility was leached more easily during the gelatinization process [30], which would reduce the transparency of soup. Therefore, the waxy rice balls prepared with flour with high solubility had a lower transparency after cooking, which was not conducive to the quality of waxy rice balls.

In addition to the external quality, the hardness, stickiness and chewiness of waxy rice balls after cooking are also concerns of consumers. It is generally believed that good-quality waxy rice balls have less hardness and adhesion and greater elasticity. Therefore, the texture properties of waxy rice balls were determined, and the results were shown in Table 4. In the different dry-milled flour particle size, it was found that the hardness of waxy rice balls decreased significantly when particle size decreased. The hardness were decreased from 194.06 g to 151.93 g and from 158.48 g to 142.88 g, respectively. There was no obvious difference in elastic properties, but the recovery value was significantly improved with the decrease of particle size, from 0.20 and 0.18 to 0.21, respectively. This indicated that the reduction of particle size could improve the quality of dry-milled waxy rice balls. When the particle size were similar to those of wet-milled, the dry-milled waxy rice balls still had a higher hardness, chewiness, elasticity and lower resilience. At the same time, it was observed that the hardness and chewiness of JWR were lower in two cultivars. Whether these subtle differences in texture characteristics will affect the taste of waxy rice balls needs further analysis through sensory evaluation.

#### 3.2.4. Sensory Evaluation, Volatile Component Analysis and Consumer Test of Waxy Rice Balls

**Figure 7:**
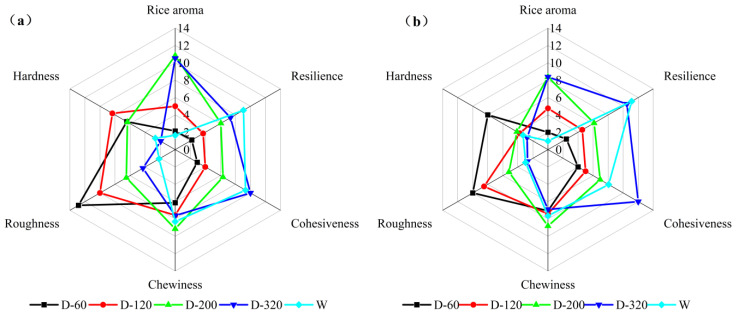
Differences in sensory evaluation of waxy rice balls. (**a**) stands for IWR and (**b**) stands for JWR.

The quality of waxy rice balls was further evaluated by descriptive analysis. Sensory attribute descriptors, definitions and reference samples were given through group discussions, as shown in Table S1 and the results were shown in Figure 7. In terms of texture characteristics, wet-milled waxy rice balls had lower hardness and stronger resilience, chewiness and cohesion. On the contrary, dry-milled rice balls with large particle size had higher hardness, worse cohesion and obvious roughness. However, its roughness and hardness decreased, cohesion and resilience increased significantly with the decrease of dry-milled flour particle size. Only when the particle size of dry-milled flour was reduced to be similar to that of wet-milled can they have similar texture scores. This indicated that reducing the particle size of dry-milled flour could significantly improve the texture of its rice balls. In addition, it was also observed that dry-milled rice balls had higher aroma scores, and the aroma score increased significantly with the decrease in particle size. This meant milling methods had a significant impact on the volatile compounds of waxy rice flour. 

Therefore, the composition and concentration of volatile compounds that may affect the aroma of waxy rice flour were determined with SPME-GC-MS, and the results were shown in Table S2. A total of 87 substances were detected and mainly divided into eight categories: aldehydes, alcohols, ketones, esters, acids, phenols, heterocyclic compounds and alkanes. It could be seen from Table S1 that with the decrease of dry-milled flour particle size, the types of volatile compounds increased from 47 and 48 to 66 and 69, respectively. This resulted in higher amounts of volatile compounds in IWR-D-320 and JWR-D-320 than those of wet-milled (46 and 48, respectively) under the same particle size range. It has been reported that aldehydes contribute greatly to the aroma of rice, hexanal [31], (E)-2-heptenal, trans-2-octenal and trans-2-nonanal [32]; octanal and nonaldehyde may contribute to the grassy and fruity fragrance. The threshold of octanal is 0.88 μg/mL, with a slight fruity aroma [33]. In IWR/JWR-D, the concentration of octanal was higher than its threshold, and it increased by 2.5 times and 7.6 times, respectively, with the decrease of particle size, which was helpful to enhance the aroma of dry-milled waxy rice flour with small particle size. Moreover, nonanal has the aroma of citrus, cucumber, floral, fresh, grassy, soapy, etc., with the threshold of 3.1 μg/mL [33]. The concentration of nonanal in all samples was significantly higher than its threshold (at least 4.6 times higher). In both groups of samples, it was observed that the concentration of nonanal increased significantly with the decrease of particle size. In IWR-D, it increased by 2.73 times, and increased by 2.2 times in JWR-D. At the same time, it could be found that the nonanal concentration of dry-milled flour were 78% and 26% higher than that of wet-milled, respectively, in the same particle size range. These differences endowed small particle size dry-milled flour with stronger rice aroma. In addition, hexanol can contribute to herbal flavor and is the most abundant volatile compound in rice, with a fairly low odor threshold [34]. In IWR-D and JWR-D, the concentration of hexanol increased significantly with the decrease of particle size by 3.55 and 1.39 times, respectively. In the same particle size range, the hexanol concentration of dry-milled flour was 3.17 and 3.14 times higher than that of wet-milled, respectively, which greatly enhanced the rice aroma of dry-milled flour. This showed that particle size reduction could enhance the rice aroma by increasing the type and content of volatile compounds in dry-milled flour. Within the same particle size range, dry-milled flour had stronger rice aroma than wet-milled. This results was consistent with the aroma scores in descriptive analysis.

**Figure 8:**
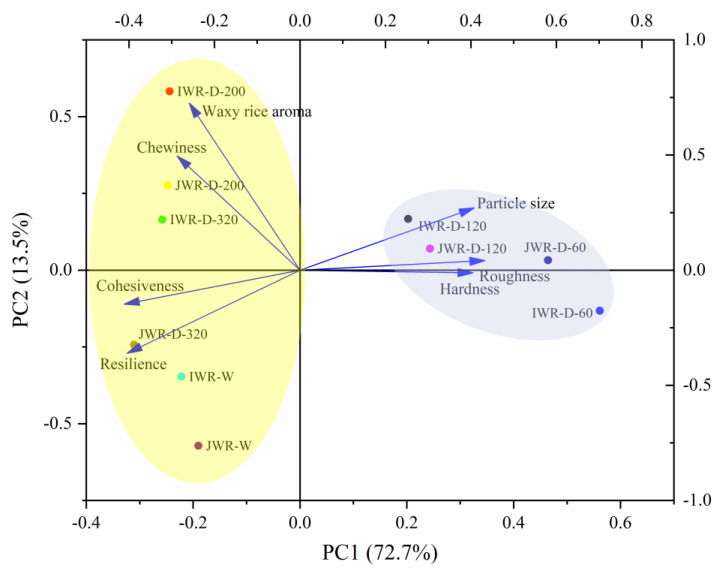
The PCA analysis of sensory evaluation.

The results of descriptive analysis were further analyzed by PCA, as shown in Figure 8. PCA analysis showed that the overall interpretation degree was 86.22%, which meant that dry-milled and wet-milled waxy rice balls could be clearly distinguished. Combined with the results of descriptive analysis, it was considered that IWR/JWR-D-60 and IWR/JWR-D-120 had higher roughness and hardness and worse cohesion and elasticity. However, the texture of dry-milled waxy rice balls increased with the decrease of particle size. The hardness and roughness of IWR/JWR-D-200 and IWR/JWR-D-320 waxy rice balls decreased while the elasticity and cohesion increased significantly. The results showed that the dry-milled waxy rice balls with small particle size had similar texture characteristics to the wet-milled, such as better chew-ability, cohesion and resilience. In addition, the rice aroma of IWR/JWR-D-200 and IWR/JWR-D-320 waxy rice balls were stronger than that of wet-milled. As determined by GC-MS, IWR/JWR-D-200 and IWR/JWR-D-320 had higher concentrations of hexanal, (E)-2-heptenal, trans-2-octenal, trans-2-nonanal, octanal, nonaldehyde and hexanol, which help to improve the rice aroma of its waxy rice balls and enhance the taste of waxy rice balls. However, there was no significant difference in the taste of waxy rice balls between two cultivars. It could be seen from Table S1 that the concentrations of Hexanal in IWR-D-200 and JWR-D-200 were 63.74 μg/mL and 65.08 μg/mL, respectively, and those in IWR-D-320 and JWR-D-320 were 73.43 μg/mL and 75.20 μg/mL, respectively, showing no significant difference. The same trend were also observed in the concentration of Nonanal and (E)-2-Heptenal.

Based on all the results, it was considered that IWR-D-200 and JWR-D-200 rice balls had suitable freeze-cracking rate and transparency and were similar to wet-milled waxy rice balls in texture and taste. Besides, it had a stronger aroma of waxy rice. Therefore, IWR-D-200 and JWR-D-200 rice balls were selected for quality comparison with wet-milled waxy rice balls under the same cooking conditions. Consumer acceptance of samples were obtained through the 9-point hedonic scales, and the results were reported in Figure 5d. In the mean value of overall liking, consumers preferred waxy rice balls prepared with IWR-D-200 and JWR-D-200, which scored around 6 points, while wet-milled waxy rice balls scored around 5 points. In order to further explore the consumers’ evaluation of waxy rice balls taste, the descriptors that consumers like or dislike the samples were counted, and the statistical results were shown in Table 5. The results showed that the chewiness and stickiness were too strong for IWR-W and JWR-W waxy rice balls. It would take more time to chew until swallowed. Therefore, consumers preferred IWR-D-200 and JWR-D-200 rice balls because of their soft texture, moderate elasticity and low chewiness. To sum up, it is feasible to prepare waxy rice balls with dry-milled waxy rice flour with a particle size of about 50 μm instead of wet-milled flour.

## 4. Conclusions

In this paper, the differences between dry-milled and wet-milled flour were discussed, and the effect of particle size on the quality of dry-milled waxy rice balls was investigated. The results showed that dry-milled waxy rice balls had a higher transparency, lower freezing crack rate and stronger rice aroma but had insufficient viscoelasticity in taste and were not smooth enough in appearance compared with the wet-milled waxy rice balls. However, the results showed that reducing the particle size of dry-milled flour could significantly improve the appearance and taste of dry-milled rice balls and enhanced the rice aroma. Sensory evaluation results showed that consumers prefer the waxy rice balls prepared with dry-milled flour in the range of 40–60 μm particle size because they were softer in texture, moderate in stickiness and chewiness and had a stronger rice aroma. Therefore, the dry-milled flour with a particle size about 40 μm to 60 μm could completely replace wet-milled to prepare waxy rice balls. This provides a theoretical support for the application of dry-milled waxy rice flour in waxy rice balls.

## Figures and Tables

**Table 1:** Particle size, damaged starch content, water holding capacity and solubility of waxy rice.

Sample	Particle Size (μm)	Damaged Starch Content (%)	Solubility (%)	Water Holding Capacity (%)
IWR-W	15.45 ± 0.11 a	6.03 ± 0.21 a	9.28 ± 0.05 a	18.13 ± 0.30 e
IWR-D-60	164.33 ± 0.88 e	9.40 ± 0.11 b	34.89 ± 0.72 b	8.79 ± 0.20 d
IWR-D-120	84.07 ± 2.63 d	16.16 ± 0.23 c	36.45 ± 0.12 c	8.54 ± 0.14 c
IWR-D-200	67.00 ± 0.10 c	19.58 ± 0.45 d	39.45 ± 0.64 d	8.38 ± 0.21 b
IWR-D-320	27.10 ± 0.20 b	26.22 ± 0.31 e	42.03 ± 0.84 e	7.92 ± 0.31 a
JWR-W	11.70 ± 0.05 a	5.70 ± 0.12 a	16.51 ± 0.04 a	16.14 ± 0.29 e
JWR-D-60	158.47 ± 1.94 e	12.03 ± 0.14 b	51.02 ± 1.05 b	6.90 ± 0.01 d
JWR-D-120	87.83 ± 3.98 d	17.29 ± 0.18 c	53.21 ± 2.13 c	6.78 ± 0.21 c
JWR-D-200	55.40 ± 0.31 c	20.36 ± 0.09 d	53.45 ± 1.26 d	6.65 ± 0.13 b
JWR-D-320	34.00 ± 0.52 b	24.64 ± 0.54 e	56.16 ± 2.54 e	6.48 ± 0.21 a

“IWR” stands for indica waxy rice; “JWR” stands for japonica waxy rice; “-D” stands for dry milled flour; “-W” stands for wet milled flour;“-number” stands for the number of sieves; Values are showed by Mean ± SD and values, different letters within a column indicate significant differences between mean values (*p* < 0.05).

**Table 2:** Fitting parameters of viscoelastic properties of waxy rice flour.

Sample	G′	G″	tanδ
IWR-W	17.79	6.61	0.368 ± 0.01 a
IWR-D-60	43.80	17.15	0.372 ± 0.00 a
IWR-D-120	44.92	16.98	0.378 ± 0.00 b
IWR-D-200	43.15	16.54	0.382 ± 0.00 c
IWR-D-320	28.11	11.68	0.415 ± 0.00 d
JWR-W	29.26	10.50	0.358 ± 0.00 a
JWR-D-60	27.19	13.79	0.509 ± 0.00 b
JWR-D-120	18.93	10.51	0.538 ± 0.03 c
JWR-D-200	18.49	10.49	0.564 ± 0.01 d
JWR-D-320	13.27	7.71	0.610 ± 0.04 e

Values are showed by Mean ± SD and values, different letters within a column indicate significant differences between mean values (*p* < 0.05).

**Table 3:** Fitting parameters of creep characteristics of waxy rice dough.

Sample	MCS (%)	MRS (%)	Recovery (%)
IWR-W	0.36 ± 0.02 c	0.14 ± 0.01 b	38.89 a
IWR-D-60	0.51 ± 0.01 d	0.21 ± 0.00 c	41.18 c
IWR-D-120	0.54 ± 0.01 e	0.22 ± 0.00 d	40.74 b
IWR-D-200	0.19 ± 0.04 b	0.15 ± 0.04 b	76.68 d
IWR-D-320	0.12 ± 0.00 a	0.11 ± 0.00 a	95.66 e
JWR-W	0.18 ± 0.01 a	0.07 ± 0.00 a	38.89 b
JWR-D-60	0.53 ± 0.04 d	0.20 ± 0.01 b	37.74 a
JWR-D-120	0.98 ± 0.01 e	0.40 ± 0.03 e	40.82 c
JWR-D-200	0.46 ± 0.02 c	0.33 ± 0.01 d	74.03 d
JWR-D-320	0.23 ± 0.02 b	0.21 ± 0.02 c	93.25 e

The value of MCS stands for maximum creep strain, the value of MRS stands for maximum recovery strain. The Recovery (%) was calculated by MRS and MCS in Formula (3). Values are showed by Mean ± SD and values, different letters within a column indicate significant differences between mean values (*p* < 0.05).

**Table 4:** Texture properties of waxy rice balls.

Sample	Hardness/g	Chewiness	Springiness	Resilience
IWR-W	92.14 ± 5.17 a	50.03 ± 2.74 a	0.85 ± 0.00 b	0.25 ± 0.02 c
IWR-D-60	194.06 ± 10.13 e	107.50 ± 4.68 e	0.95 ± 0.01 e	0.20 ± 0.02 a
IWR-D-120	178.15 ± 1.03 d	87.43 ± 1.86 b	0.84 ± 0.02 a	0.20 ± 0.01 a
IWR-D-200	158.81 ± 8.02 c	92.13 ± 2.25 c	0.90 ± 0.00 d	0.21 ± 0.02 b
IWR-D-320	151.93 ± 2.81 b	99.65 ± 2.59 d	0.87 ± 0.02 c	0.21 ± 0.02 b
JWR-W	83.38 ± 5.23 a	86.22 ± 2.06 c	0.87 ± 0.01 a	0.31 ± 0.01 d
JWR-D-60	158.48 ± 6.24 e	96.16 ± 3.58 e	0.90 ± 0.00 b	0.18 ± 0.01 a
JWR-D-120	152.84 ± 5.70 d	72.39 ± 2.06 a	0.90 ± 0.01 b	0.19 ± 0.00 b
JWR-D-200	149.32 ± 3.13 c	73.10 ± 0.23 b	0.90 ± 0.03 b	0.19 ± 0.02 b
JWR-D-320	142.88 ± 4.32 b	87.45 ± 0.23 d	0.90 ± 0.02 b	0.21 ± 0.02 c

Values are showed by Mean ± SD and values, different letters within a column indicate significant differences between mean values (*p* < 0.05).

**Table 5:** Descriptors of samples in consumer testing.

Sample	Consumer Likes/Dislikes Reasons (Word Frequency)
IWR-D-200	Soft texture (13), Moderate elasticity (6), Softer (4), Less chewy (5);
IWR-W	Harder (6), Good chewiness (8), Too elasticity (5), Strong stickiness (10), More delicate (3);
JWR-D-200	Softer texture and less hardness (10), more waxy (6), Moderate stickiness (11), Easy Chew (3), Moderate elasticity (4), Moderate hardness (1);
JWR-W	Chewable (11), good elasticity (7), smooth and delicate (3), Too sticky (5), Too chewy (4);

The number represents the occurrence frequency of the descriptor.

## Data Availability

Data are contained within the article and Supplementary Materials.

## Supplementary Materials

The following supporting information can be downloaded at: https://www.mdpi.com/article/10.3390/foods12020280/s1, Table S1: Sensory descriptors, definitions and reference samples of waxy rice balls; Table S2: Volatile compounds in the waxy rice flour.
