# Supplementary material for: The Possibility of Replacing Wet-Milling with Dry-Milling in the Production of Waxy Rice Flour for the Application in Waxy Rice Ball
Source: Foods. 2023 Jan 6;12(2):280. doi: 10.3390/foods12020280 (PMC9858382; doi:10.3390/foods12020280)
Supplement: Supplementary file 1 [file foods-12-00280-s001.zip › foods-2119699-supplementary.pdf]

Table S1. Sensory descriptors, definitions and reference samples of waxy rice balls

Sensory descriptors	Evaluation definitions	Reference samples
Hardness	Take the waxy rice ball skin of the same size and record the force required to bite the same section with the molars. The higher the force, the higher the score	Small waxy rice balls cooked for 5 minutes at 800w power (4 points)<br>Small waxy rice balls cooked for 3 minutes at 800w power (8 points)<br>Chopped rice cakes cooked for 3 minutes at 800w power (10 points)
Cohesiveness	Take the waxy rice ball skin of the same size and chew it 5 times. The smaller/more pieces of the sample, the worse the cohesion and the lower the score; the larger/less the number of pieces, the better the cohesion and the higher the score.	Small waxy rice balls cooked for 3 minutes at 800w power (14 points)<br>Chopped rice cakes cooked for 10 minutes at 800w power (8 points)<br>Chopped rice cakes cooked for 15 minutes at 800w power (4 points)
Chewiness	Take the waxy rice ball skin of the same size, record the chewing times until natural swallowing, and convert it into score. The more times, the higher the score.	Chopped rice cakes cooked for 3 minutes at 800w power (8 points)
Resilience	Take the waxy rice ball skin of the same size, squeeze the sample between the tongue and the upper jaw, and feel the degree of resilience of the sample. The greater the deformation recovery, the greater the elasticity of the sample, and the higher the score.	Internal standard
Roughness	Take the waxy rice balls of the same size, and feel the maximum roughness of the sample during chewing. The higher the roughness, the higher the score.	Internal standard
Waxy rice aroma	Taste the sample and feel the strongest waxy rice aroma during chewing	Small waxy rice balls cooked for 3 minutes at 800w power (3 points)<br>Fresh raw rice (10 points)

Table S2. Volatile compounds in the waxy rice flour

Number	Compounds	Content (μg/mL)									
		IWR-D-60	IWR-D-120	IWR-D-200	IWR-D-320	IWR-W	JWR-D-60	JWR-D-120	JWR-D-200	JWR-D-320	JWR-W
**Alcohols**											
A1	1-Decanol, 2-hexyl-	—	—	0.24±0.01a	1.24±0.02b	—	—	—	1.12±0.02a	1.31±0.03b	—
A2	1-Heptanol	0.35±0.01a	2.13±0.01b	2.21±0.10c	2.79±0.03d	4.91±0.02e	—	3.11±0.01a	3.12±0.00a	3.95±0.03b	6.22±1.02c
A3	1-Hexanol	1.39±0.02a	2.53c	2.60d	4.94e	1.56±0.01b	8.64±0.04b	9.81±0.03c	10.87±0.08d	11.98±1.13e	3.82±0.02a
A4	1-Octanol	1.87±0.12a	2.74±0.04b	2.94±0.01c	5.9±0.02d	—	1.56±0.03a	2.92±0.04b	3.77±0.32c	4.85±0.78d	—
A5	1-Pentanol	8.52±0.01c	9.24±0.02d	8.15±0.02b	7.88±0.05a	28.57±1.21e	9.82±1.12a	10.76±0.13c	—	10.02±0.15b	28.20±1.24e
A6	2,3-Butanediol	—	0.76±0.01a	1.63±0.04b	1.95±0.01c	—	—	0.85±0.05b	0.81±0.12a	1.28±0.14c	—
A7	2,3-Butanediol, [R-(R*,R*)]-	2.05±0.14c	—	0.56±0.05a	0.79±0.11b	—	—	—	0.39±0.12a	0.55±0.05b	—
A8	2-Propanol, 1-(2-methoxy-1-methyl ethoxy)-	0.74±0.05c	0.10±0.01a	0.11±0.02a	—	—	—	—	0.24±0.01a	0.28±0.03b	—
A9	2-Propanol, 1,1'-oxybis-	3.43±0.18c	0.37±0.01b	—	0.21±0.02a	—	4.33±1.02d	0.57±0.07c	0.34±0.01a	0.47±0.02b	—
A10	3-Buten-2-ol, 2-methyl-	0.78±0.03c	0.04±0.02a	—	—	0.36±0.05b	1.49±0.06c	0.14±0.02a	—	—	0.28±0.05b
A11	9-Octadecen-1-ol, (Z)-	3.55±0.02b	—	—	0.31±0.09a	—	—	—	—	1.66	—
A12	Benzyl alcohol	1.10±0.00c	0.33±0.08a	0.71±0.01b	1.11±0.10c	2.67±0.09d	0.71±0.03b	0.98±0.07c	0.48±0.01a	6.52±1.12e	2.87±0.13d
A13	trans-2-Dodecen-1-ol	—	1.26	—	—	—	—	0.95	—	—	—
**Esters**											
B1	.delta.-Nonalactone	—	—	—	—	0.32	—	0.93±0.02a	—	1.89±0.13b	—
B2	2(3H)-Furanone, 5-butyldihydro-	—	0.09±0.01a	—	0.17±0.03b	1.58±0.12c	—	0.14±0.01c	0.08±0.01a	0.12±0.03b	1.49±0.11d
B3	2(3H)-Furanone, 5-ethyldihydro-	—	0.78±0.07b	0.23±0.01a	—	7.90±0.04c	—	—	0.55±0.02a	—	7.00±1.171b
B4	2(3H)-Furanone,	—	—	—	—	0.82	—	—	—	—	0.91

	dihydro-5-methyl-										
B5	2,2,4-Trimethyl-1,3-pentanediol diisobutyrate	1.84±0.13b	0.95±0.02a	—	—	—	—	—	1.69	—	—
B6	2H-Pyran-2-one, tetrahydro-6-methyl-	0.95±0.05a	—	—	—	1.45±0.09b	—	—	—	—	1.30
B7	Acetic acid, phenyl ester	—	—	—	0.19	—	0.88±0.04b	—	—	0.18±0.03a	—
B8	Butanoic acid, methyl ester	—	—	—	1.46	—	—	—	—	0.50	—
B9	Formic acid, hexyl ester	6.26±0.14c	—	1.28±0.02a	1.32±0.03b	—	7.25±1.15c	—	1.41±0.03a	2.46±0.04b	—
B10	Hexadecanoic acid, methyl ester	—	0.64±0.02c	0.46±0.05a	0.54±0.10b	1.71±0.18d	—	0.44±0.10a	1.52±0.03d	1.14±0.04c	0.76±0.05b
B11	Hexanedioic acid, dimethyl ester	7.73±0.28b	—	—	0.48±0.03a	—	12.89±2.13 b	—	—	3.04±0.13a	—
B12	Isopropyl myristate	—	—	—	—	0.17	—	—	0.25±0.01a	—	0.82±0.06b
B13	Methyl methacrylate	—	0.34±0.03b	0.25±0.08a	0.52±0.08c	—	2.320.13c	0.21±0.02a	0.22±0.01a	0.43±0.02b	—
B14	Methyl tetradecanoate	0.81±0.09b	—	—	0.27±0.03a	—	—	—	0.17±0.01a	0.19±0.03b	—
B15	Nonyl chloroformate	—	—	—	1.97	—	—	—	—	3.16	—
B16	Octanoic acid, methyl ester	—	—	—	0.80±0.01a	1.68±0.12b	1.20±0.06b	—	—	—	1.07±0.02a
B17	Octyl chloroformate	—	—	—	2.92	—	—	—	2.03	—	—
B18	Pentanedioic acid, dimethyl ester	1.45±0.14d	0.18±0.02a	—	0.84±0.07b	0.93±0.04c	—	0.59±0.01a	0.66±0.12b	6.76±1.27d	1.20±0.13c
**Acids**											
C1	Acetic acid	—	1.04	—	—	—	—	—	—	—	5.92
C2	Butanoic acid	—	0.40±0.01a	—	—	36.35±1.17b	—	—	—	—	3.46
C3	Heptanoic acid	—	0.22±0.00a	0.23±0.02a	0.34±0.04b	7.96±0.18d	—	0.26±0.02a	0.32±0.04b	0.38±0.03c	9.67±2.13d
C4	Hexanoic acid	1.66±0.12a	4.39±0.41b	4.46±0.15c	5.56±0.12d	64.64±1.17e	1.68±0.31a	5.49±0.27b	5.97±0.15c	7.05±1.47d	69.03±3.15e

C5	Hexanoic acid, anhydride	—	—	—	—	2.75	—	—	1.32±0.02a	—	3.69±0.94b
C6	n-Decanoic acid	—	1.69±0.14c	0.32±0.02a	1.57±0.08b	2.50±0.21d	2.02±0.03d	1.73±0.04a	1.92±0.12b	2.05±0.06c	—
C7	Nonanoic acid	—	4.60±0.12b	3.05±0.21a	4.59±0.12b	10.10±1.12c	—	6.04±0.06b	7.47±0.15c	5.58±0.02a	13.64±1.23d
C8	Octanoic acid	—	1.07±0.01b	0.94±0.03a	1.59±0.02c	14.83±1.12d	0.99±0.12b	0.59±0.03a	1.23±0.17c	1.66±0.23d	18.55±1.29e
C9	Pentanoic acid	—	0.64±0.03b	0.55±0.12a	1.03±0.10c	15.06±1.23d	—	0.81±0.12b	0.40±0.06a	1.16±0.15c	17.69±1.32d
**Ethers**											
D1	Octaethylene glycol monododecyl ether	2.25±0.17d	0.24±0.01b	0.17±0.02a	0.48±0.05c	0.49±0.02c	2.53±0.07d	—	0.47±0.11b	0.10±0.03a	0.92±0.04c
D2	Pentaethylene glycol monododecyl ether	—	—	—	0.16	—	1.70±0.13c	—	0.07±0.00a	0.20±0.01b	—
**Aldehydes**											
E1	2,4-Heptadienal, (E,E)-	—	—	—	—	—	0.88±0.06c	0.18±0.01a	—	0.28±0.07b	—
E2	2,4-Nonadienal, (E,E)-	—	0.45±0.02a	0.73±0.01b	1.30±0.21c	1.45±0.05d	—	0.52±0.10a	0.73±0.12b	1.11±0.05d	1.01±0.02c
E3	2-Heptenal, (E)-	0.53±0.05a	2.99±0.14b	4.48±0.17c	5.26±0.52d	4.94±0.26e	0.21±0.02a	3.58±0.00b	4.74±0.12d	4.90±0.56e	3.60±0.02c
E4	2-Hexenal	—	—	0.31±0.08a	0.36±0.03b	—	0.57±0.08a	—	—	1.80±0.06b	—
E5	2-Hexenal, (E)-	0.14±0.05a	—	0.40±0.02b	1.03±0.07c	2.04±0.09d	—	0.70±0.02a	0.93±0.12b	1.07±0.13c	—
E6	2-Nonenal, (E)-	2.09±0.13a	4.37±0.18d	5.82±0.51c	8.89±0.23d	—	1.20±0.11a	2.52±0.17b	5.36±0.05c	7.81±1.12d	—
E7	2-Octenal, (E)-	1.71±0.04a	—	1.92±0.03b	8.66±1.10c	—	1.71±0.03a	4.80±0.15b	5.80±0.17c	7.04±1.32d	—
E8	Benzaldehyde	3.54±0.12a	4.34±0.08b	7.46±0.14c	9.02±1.12d	16.00±0.16e	4.20±0.23a	6.04±1.14b	8.32±0.13c	10.72±0.58d	16.81±1.45e
E9	Butanal	2.48±0.12b	—	—	—	1.38±0.05a	1.59±0.13b	—	—	—	1.42±0.03a
E10	Butanal, 3-methyl-	—	3.96±0.15a	—	4.68±0.23b	5.15±0.14c	—	5.26±1.18a	—	—	10.58±2.15b
E11	Decanal	—	—	—	2.55±0.12a	4.18±0.78b	—	2.90±0.36b	1.88±0.15a	—	3.94±0.51c
E12	Heptanal	—	4.10±0.18a	5.62±0.12b	5.87±0.45c	7.24±1.12d	—	4.86±0.16a	5.52±1.25b	5.71±0.17c	8.27±1.26d
E13	Hexanal	49.85±1.23a	51.79±2.14b	63.74±5.23c	73.43±2.74d	45.29±3.16e	52.21±6.12b	62.08±3.15c	65.08±2.48d	75.20±9.54e	42.10±4.21a
E14	Nonanal	15.21±0.12a	17.15±0.61b	30.30±2.35c	41.56±1.56d	23.39±2.45e	14.23±0.50a	16.50±1.45b	28.61±2.47d	31.30±5.32e	24.84±3.12c

E15	Octanal	1.43±0.02a	2.66±0.41b	3.54±0.32c	3.63±0.12d	5.95±1.17e	0.57±0.04a	4.03±0.17b	4.39±0.13d	4.36±0.17c	5.71±0.16e
E16	Pentanal	1.24±0.05a	—	4.92±0.07b	5.64±1.11c	—	3.52±1.10a	—	6.56±0.76c	4.11±0.12b	—
E17	Tetradecanal	1.20±0.02a	—	—	2.08±0.56b	—	1.08±0.01a	—	—	2.38±0.15b	—
**Ketones**											
F1	2(3H)-Furanone, dihydro-4-methyl-5-pentyl-	—	—	—	—	0.17	—	—	—	—	0.12
F2	2,3-Octanedione	0.54±0.02a	0.65±0.06b	0.96±0.12c	1.62±0.23d	—	0.42±0.01a	0.41±0.00a	0.62±0.12b	0.76±0.03c	—
F3	2-Butanone	0.21±0.04a	0.87±0.12d	0.45±0.07c	0.25±0.03b	2.53±0.56e	0.41±0.02a	0.60±0.12b	0.84±0.14c	—	1.95±0.13d
F4	2-Heptanone	2.40±0.04a	3.34±0.10b	6.24±0.85e	5.05±0.12d	3.46±0.01c	2.70±0.01a	4.73±0.32b	9.38±1.45e	6.68±0.56d	5.01±0.51c
F5	2-Nonanone	—	0.17±0.02a	0.46±0.12b	0.90±0.17c	—	—	0.23±0.13a	0.65±0.09b	0.79±0.05c	—
F6	2-Octanone	0.15±0.03a	—	1.57±0.11b	—	—	0.10±0.02a	1.31±0.11b	1.92±0.21c	—	—
F7	3-Nonen-2-one	0.10±0.03a	—	0.26±0.04b	0.47±0.02c	—	0.43±0.02a	0.85±0.13b	1.21±0.10c	1.52±0.31d	—
F8	3-Octanone	0.85±0.03b	—	—	0.34±0.05a	—	—	—	—	0.62	—
F9	3-Octanone, 2-methyl-	—	—	—	—	1.73	—	—	—	—	5.08
F10	3-Octen-2-one	—	—	—	9.44±1.12b	2.30±0.03a	0.25±0.02a	—	—	3.69±0.45c	2.83±0.13b
F11	5,9-Undecadien-2-one, 6,10-dimethyl-, (E)-	0.35±0.01a	0.76±0.06b	0.69±0.10c	1.55±0.23d	2.54±0.13e	0.89±0.01a	1.00±0.12b	1.49±0.03c	1.65±0.07d	2.40±0.13e
F12	5-Hepten-2-one, 6-methyl-	—	2.75±0.24a	3.46±0.23b	3.75±0.14c	—	—	—	—	—	—
F13	6-Methyl-3,5-heptadiene-2-one	0.23±0.03a	0.34±0.04b	0.39±0.01c	0.38±0.02c	1.55±0.07d	0.41±0.02a	0.52±0.01b	0.56±0.04c	0.80±0.10d	1.67±0.21e
F14	Acetophenone	0.28±0.01a	0.61±0.10d	0.42±0.03b	0.51±0.02c	1.32±0.12e	0.12±0.00a	0.66±0.07c	0.60±0.01b	1.14±0.04d	1.32±0.07e
F15	Bicyclo[3.1.1]heptan-2-one, 6,6-dimethyl-, (1R)-	—	—	—	0.36	—	—	—	0.25±0.02a	0.44±0.01b	—
F16	CH3C(O)CH2CH2OH	—	—	—	—	0.86	—	—	—	—	1.51
**Phenols**											
G1	2,4-Di-tert-butylphenol	—	0.63±0.03b	0.32±0.01a	0.71±0.04c	—	—	—	0.57±0.03a	0.66±0.02b	—
G2	Phenol	0.60±0.03e	0.09±0.00a	0.11±0.01b	0.13±0.03c	0.20±0.01d	0.46±0.03d	—	0.12±0.03b	0.10±0.01a	0.41±0.00c

**Heterocycles**											
H1	2-n-Butyl furan	0.89±0.02a	1.16±0.12b	1.40±0.14c	1.89±0.02d	1.71±0.05e	1.21±0.10a	1.47±0.12b	1.98±0.05d	1.92±0.23c	1.47±0.05b
H2	Furan, 2-pentyl-	7.62±0.15b	7.55±0.03a	8.15±0.16c	9.44±0.16e	9.35±0.05d	10.01±0.00a	10.13±0.00d	10.03±0.01b	10.11±0.03c	13.08±1.23e
H3	Furan, 2-propyl-	1.10±0.01c	—	0.14±0.03a	0.18±0.00b	—	—	—	—	0.28	—
H4	Naphthalene	0.28±0.01b	0.48±0.03a	0.23±0.00a	0.78±0.06e	0.54±0.11d	0.43±0.02a	0.57±0.00c	0.56±0.01c	0.46±0.06b	0.75±0.04d
H5	Naphthalene, 1-methyl-	0.02±0.00a	0.13±0.01b	0.14±0.01b	0.58±0.12c	—	—	—	—	1.35	—
H6	Naphthalene, 2,3-dimethyl-	—	—	—	0.21	—	0.11±0.00a	—	—	0.10±0.02a	—
H7	Naphthalene, 2,7-dimethyl-	0.39±0.02b	—	—	0.17±0.03a	—	0.32±0.06b	—	—	0.28±0.02a	—
H8	Naphthalene, 2-methyl-	0.08±0.01a	0.12±0.03b	0.13±0.01b	0.38±0.03c	—	—	0.22±0.01a	0.30±0.01b	0.40±0.00c	—
**Alkanes**											
I1	D-Limonene	3.71±0.03e	3.42±0.01d	3.32±0.03c	3.29±0.02b	2.96±0.09a	2.56±0.02a	2.76±0.31b	2.92±0.02c	3.71±0.08e	2.96±0.04d
I2	Styrene	—	0.55±0.02a	1.26±0.13b	1.62±0.45c	—	—	1.06±0.01b	0.57±0.03a	1.71±0.23c	—
**Total number of detected substances**		47b	49c	59d	66e	46a	48a	52b	53c	69d	48a

Values are showed by Mean ± SD and values, different letters within a column indicate significant differences between mean values ($p < 0.05$).
